# The bereaved parent

**DOI:** 10.7554/eLife.49932

**Published:** 2019-07-08

**Authors:** Edward Wallace

**Affiliations:** Institute for Cell BiologyUniversity of EdinburghEdinburghUnited Kingdom

**Keywords:** scientist and parent, careers in science, diversity, grief, research culture, support

## Abstract

The impacts of grief are long-lasting, but support from colleagues provides some relief.

My daughter Sadie Ruth was stillborn in November 2017. My wife and I held our daughter in our arms and cried endless tears, but never heard Sadie cry for herself. Two days later, still in shock, emerging from the registrar’s office with the certificate of stillbirth in my hand, my phone rang: the Wellcome Trust was calling to tell me that I had been awarded a Sir Henry Dale Fellowship to start my own lab at the University of Edinburgh. I had my dream job, studying how dynamic messenger RNA processing allows fungal cells to adapt to a changing environment. This extreme contrast still makes my head spin: the worst thing that could happen to my family in the same week as the best thing that could happen to my career.

Many colleagues have since told me stories of their own tragedies across spectra of losing children, losing pregnancies, struggling with fertility, and divorce. Collectively, these tragedies are common and challenge a scientist’s career in ways very different from the challenges of raising children. Parents look after their children every day, waking to feed and comfort them, taking them to school, parties, and so on. Bereaved parents live every day without their child, a loss that feels invisible to others. I celebrate my colleagues’ beautiful babies as they arrive – so many babies – and shed more tears for my own daughter, who they will never meet.

My colleagues and institution were amazingly supportive. A kind university administrator converted my booked parental leave into compassionate leave on full pay and arranged all the paperwork without troubling me. Some colleagues offered to come to Sadie Ruth’s funeral, although we kept it a family affair. Dozens from Edinburgh and around the world sent flowers, wrote heartfelt notes, and passed on the news so I didn’t have to. They listened to me talk, replied to my emails, and checked in with me over the weeks and months afterwards. My friends and colleagues taught me what it means to be truly supportive with their words and actions, and now I have to pay that strength forward. Science is a community endeavour, and I’m proud to work in such a supportive community.

My career in science helped me to survive the loss. At the time, receiving my fellowship was a source of relief rather than happiness. Having spent months in an increasing state of panic while expecting a baby, applying for jobs, and seeing my contract end date loom ever closer, the new fellowship gave me the stability to breathe. For a few weeks I could focus on looking after my wife and myself, arranging the funeral, and accommodating to being devastated, exhausted, and barely functional. Then a particularly kind colleague hosted me for a two-month visit to his lab in Paris, where the change of scene and routine was another relief. The science was exciting as well, and in May 2019 we submitted our first joint paper as co-corresponding authors. These people and projects gave me reasons to get up in the morning, when that was a struggle.

I returned to Edinburgh ready to recruit a lab manager, occupy my new space, and start building my group. Several grant applications and PhD recruitment rounds later, we have six people in the team and we are all learning from each other. It is such a pleasure to work with great people – thoughtful scientists and kind human beings – attacking interesting questions. I have told my lab about my daughter, because I don’t have the strength to hide it, because if I’m crying in my office I’d rather they knew why, and because loss is normal so we may as well admit it and be compassionate.

Still 18 months afterwards, I’ve barely recovered the energy to do my job the way I want to.

I have learned so much more than I wanted to about grief and how to respond to it. Facing up to loss is hard, whether our own or someone else’s, and any thoughtful attempt to do so will be appreciated. Every message saying “I don’t know what to say, but I’m thinking of you”, or “I’m sorry for your loss”, meant the world. The messages that reminded my wife and I that we were parents, that we had produced a child and we would miss her and the person she could have grown into, were vital. When people ask about Sadie’s name, and talk about her as a real person, it reminds us that she ever existed, and this is the biggest lesson I’ve learned in speaking to others about loss.

All of this I had to process while attempting to be a world-class scientist with an absorbing full-time job. Still 18 months afterwards, I’ve barely recovered the energy to do my job the way I want to. Guy Tanentzapf puts it well: “Doing science is challenging enough when everything is going great in a person’s life but becomes next to impossible for those facing life struggles. I came to realise that the first questions we as colleagues should ask someone who’s struggling with their science is ‘are you ok?’ & ‘can I help?’ Also we should try & think about what they may be going through.”

Fundamentally, losing my daughter tested my belief in the value of science and the predictability of the world. Nothing we or the doctors knew would likely have changed my daughter’s fate, and after an investigation we still have no diagnosis. The UK’s National Health Service does not even collect detailed statistics on births that could prevent other families from suffering like ours. In my lab, we test the probabilistic survival of a proportion of yeast cells under severe stress, and accept that we will not understand the sources of this random survival. In family life, this randomness is unacceptable, incomprehensible, and cruel.

## Note

This Feature Article is part of the Scientist and Parent collection.

